# A two-person neuroscience perspective on parent-infant dyadic expansion of consciousness

**DOI:** 10.3389/fnhum.2026.1727030

**Published:** 2026-04-15

**Authors:** Giorgia Procissi, Elena Capelli, Beatrice Riva, Martina Collura, Lucia Billeci, Valentina Riva, Ed Tronick, Livio Provenzi

**Affiliations:** 1 National Research Council, Institute of Clinical Physiology, Pisa, Italy; 2 Department of Brain and Behavioral Sciences, University of Pavia, Pavia, Italy; 3 Child Psychopathology Unit, Scientific Institute IRCCS E. Medea, Bosisio Parini, Italy; 4 Departments of Psychiatry and Pediatrics, University of Massachusetts Chan Medical School, Worcester, MA, United States; 5 Developmental Psychobiology Lab, IRCCS Mondino Foundation, Pavia, Italy

**Keywords:** dyadic expansion of consciousness, EEG, hyperscanning, mutual regulation model, still-face paradigm

## Abstract

Caregiver-infant interaction represents the space where development happens through time. According to the mutual regulation model (MRM) by Tronick, meaning-making, emotion regulation, and stress resilience all emerge from the complex fabric of caregiver-infant interaction. Within this model, the dyadic expansion of consciousness (DEC) identifies how adult caregivers and infants co-create an expanded state of consciousness characterized by greater complexity through reciprocal interactions of their individual states of consciousness and alternating phases of matching, mismatching, and reparation. The well-validated Face-to-Face Still-Face paradigm (FFSF), by introducing experimental manipulations of caregiver's interactive availability, represents a reliable procedure to investigate these early forms of socio-emotional and socio-cognitive exchanges. Nonetheless, there is a general lack of studies investigating and providing measures of DEC. Recent advancements in the developmental neuroscience field (i.e., hyperscanning protocols) hold promises to provide renewed interest in studying DEC by exploring the dyadic co-regulation of inter-brain coupling and uncoupling from a caregiver-infant perspective. By employing diverse emerging metrics of neural coupling, researchers can investigate, using unprecedented neuroscientific approaches, how the behavioral and neural activity of each interactive partner may lead to the emergence of a “two-brained system” capable of producing dyadic meanings through dynamically synchronized and resonating individual brain networks. In the present contribution, we highlight how developmental hyperscanning research can be beneficial to our comprehension of the early mutual regulation processes occurring in caregiver-infant dyads.

## 1 Introduction

Caregiver-infant interaction constitutes the primary context in which early human development takes place, offering a rich and dynamic environment that supports cognitive, emotional, and social growth from the earliest stages of life. As the core primary setting through which infants engage with their proximal and distal world, these interactions have become a central focus across multiple fields of developmental science, including psychology, neuroscience, and pediatrics. A growing body of research has documented the crucial role of caregiver-infant interactions in shaping foundational developmental outcomes such as attachment (Beebe et al., 2000; Braungart-Rieker et al., 2014), language acquisition (Endevelt-Shapira et al., 2024; Masek et al., 2021), and regulatory skills (Feldman et al., 1999; Noe et al., 2015). By examining these interactions, developmental scientists not only advance theoretical understanding but also inform the design of evidence-based interventions aimed at promoting healthy development in early childhood (Bornstein and Esposito, 2023; Giusti et al., 2018; Montirosso et al., 2025).

Within this broader framework, the concept of dyadic expansion of consciousness (DEC), introduced by Tronick through the mutual regulation model (MRM; Gianino and Tronick, 1988), provides a compelling theoretical lens for investigating how caregivers and infants jointly shape each other's emotional and cognitive experiences. Through cycles of matching, mismatch, and reparation, dyadic interaction allows the co-construction of meaning and affect, whereby both members of the dyad participate in the creation of a shared emotional and cognitive reality, a sense of themselves in the world (Tronick et al., 1998). Despite its theoretical appeal, MRM and DEC have received limited empirical investigation, largely due to the methodological challenges involved in capturing the dynamic, real-time interplay between two interacting individuals.

Recent advances in two-person relational neuroscience (De Felice et al., 2025), specifically inter-brain hyperscanning, offer new opportunities to empirically examine these processes. Hyperscanning is a neuroscience methodology that allows for the simultaneous recording of brain activity from two or more individuals engaged in real-time social interaction. In the context of caregiver-infant interactions, this approach holds promise for illuminating the neural underpinnings of mutual regulation, intersubjectivity, and the dyadic expansion of consciousness. This paper explores the application of hyperscanning techniques, especially the EEG-based approach, to early developmental interactive contexts/interactions, with the goal of advancing our understanding of the shared neurobiological processes that support early relational experiences.

## 2 The mutual regulation model

The MRM (Banella et al., 2018; Cohn and Tronick, 1987) offers a foundational theoretical framework for understanding parent–infant interaction from the earliest moments of interpersonal engagement. Grounded in principles of non-linear dynamic systems theory (Sander, 1995; Von Bertalanffy, 1952, 1968), the MRM emphasizes the centrality of behavioral and affective co-regulatory processes between caregivers and infants (Beeghly et al., 2011; Provenzi et al., 2016a). These early interactions are thought to scaffold the development of shared intentionality and meaning-making, thus playing a critical role in relational and socio-emotional development (Banella et al., 2018).

According to the MRM, infants possess innate “self-organizing neurobehavioral capacities” that enable them to modulate their behavioral states and begin to interpret their experiences (Banella et al., 2018). Although these self-regulatory capacities are still immature in early infancy, they are supported and shaped through external regulation provided by the caregiver, forming a dyadic system of mutual co-regulation. In this system, caregiver and infant are understood as a single dynamic system in which both partners engage in continuous exchanges of affective signals, behavioral cues, and cognitive inputs. This dynamic reciprocity is conceptualized through the notion of “dyadic regulatory systems”—psychobiological and communicative mechanisms that jointly contribute to achieving mutual regulation, enabling infants to maintain optimal central nervous system arousal (Banella et al., 2018; Wass et al., 2024). In this context, behavioral contingency—the sequential, ‘step-by-step' coordination and mutual prediction between partners—serves as a functional prerequisite (Beebe et al., 2024). Although behavioral contingency provides the foundational scaffolding for infant development, the MRM perspective shifts the focus toward the dyadic system as a whole.

Within this framework, each member of the dyad influences, and is influenced by the other in real time, forming a single self-organizing system. A frequently used metaphor to describe these moment-to-moment exchanges is that of a dancing couple: their coordinated (but not perfectly choreographed) movements reflect the micro-temporal rhythms of interaction observed in early caregiver–infant relationships (Provenzi et al., 2018; Tronick and Reck, 2009; Wass et al., 2024).

Mutual regulation is considered successful when caregiver and infant together create, communicate, and integrate emotionally meaningful experiences, achieving moments of synchrony and fostering what has been described as implicit relational knowing (Fonagy, 2015). These experiences allow infants to organize their internal states and begin constructing a coherent understanding of themselves in relation to others. The caregiver–infant interaction thus gives rise to an emergent, co-created dyadic state that forms the foundation for future relational patterns (Fonagy, 2015; Tronick, 2003).

Importantly, the MRM acknowledges that ruptures and mismatches are a natural and frequent component of typical social interactions. Rather than signaling dysfunction, these temporary misalignments are expected and developmentally normative. In fact, the capacity of the dyad to repair such interactive disruptions through mutual co-regulation is viewed as particularly critical to development (Reck et al., 2023; Tronick, 2017). Tronick has underscored that these everyday cycles of mismatch and repair help foster infants' regulatory resilience by providing opportunities for successful resolution of micro-stressors within a supportive relational context (DiCorcia and Tronick, 2011). This process matures significantly over time: longitudinal evidence indicates that co-regulation patterns accelerate between 12 and 24 months, as infants develop age-dependent skills such as joint attention and shared meanings (Aureli et al., 2022). In this sense, the caregiver acts as a scaffolding partner who supports the infant's emerging capacity to self-regulate, to cope with and adapt to interactive challenges (Wass et al., 2024).

## 3 A paradigm of choice to study interactive reparations

**Figure 1:**
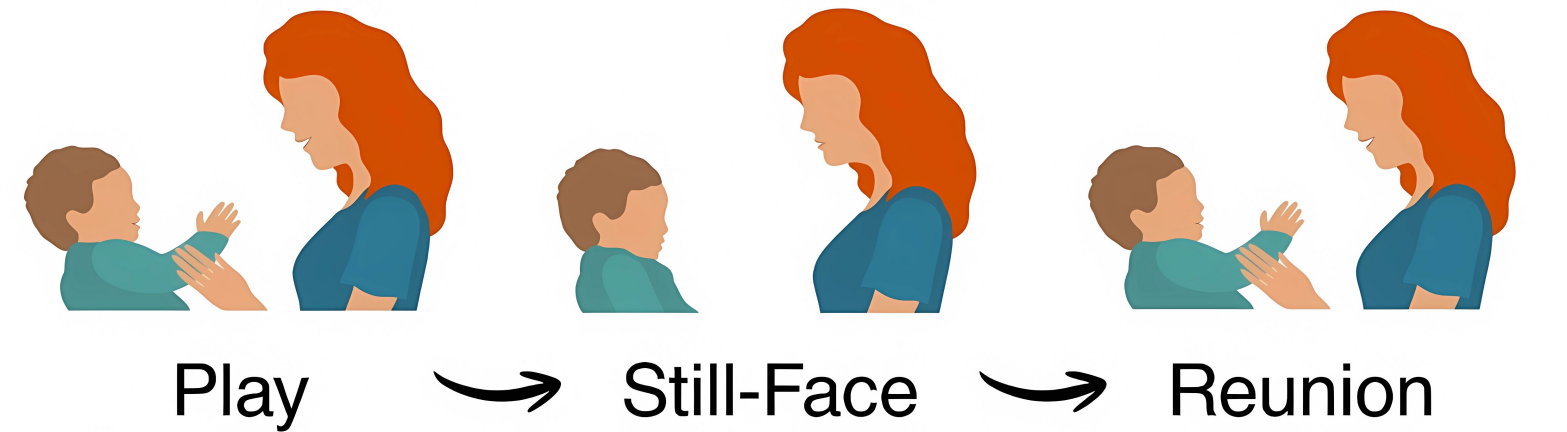
Illustration of the three episodes of the face-to-face still-face paradigm (FFSF; Tronick et al., 1978). During the Play Interaction episode, the caregiver engages with the infant. During the Still-Face Perturbation, the caregiver maintains a neutral, unresponsive expression while maintaining eye contact. During the Reunion episode, normal face-to-face interaction is resumed.

A key methodological procedure developed by Tronick to empirically investigate mutual regulation processes in early development is the Face-to-Face Still-Face paradigm (FFSF; Tronick et al., 1978). In the FFSF researchers experimentally manipulate caregiver availability to contingently respond to the infant, which in turn allows to highlight infant's sensitivity during an interactive rupture, regulatory responses, and coping mechanisms. This paradigm underscores the central role of social connectedness and dyadic regulation in early relational experiences. The standard FFSF procedure consists of three consecutive 2-min episodes (see Figure 1): (1) a baseline face-to-face interaction (Play), (2) a period of maternal unresponsiveness while maintaining eye contact (Still-Face), and (3) the resumption of interactive behavior (Reunion). These episodes provide a structured framework for observing dynamic processes such as affective matching, behavioral synchrony, and dyadic reparation (Provenzi et al., 2018).

Matching refers to the simultaneous expression of shared affective or behavioral states between caregiver and infant (Moore and Calkins, 2004). However, matching is not a static ideal but part of a dynamic cycle involving rupture, repair, and re-matching. Reparation, defined as the process of transitioning from mismatch to restored synchrony, is crucial for developing adaptive interaction strategies and regulatory competence (DiCorcia and Tronick, 2011). Dyads characterized by higher rates of reparation (Montirosso et al., 2014) and the greater frequency of reparative events during the Play episode have been linked to infants' emotional responses in subsequent Still-Face and Reunion episodes (Provenzi et al., 2015).

Disruption in reparation is associated with developmental risk. For example, dyads involving depressed mothers demonstrate lower reparation rates and require more time to resolve mismatches (Reck et al., 2011). Similarly, infants with reduced physiological regulation engage less in reparative behavior. This can be measured by an absence of vagal tone suppression, the adaptive withdrawal of parasympathetic activity that typically occurs during stressful social episodes (Provenzi et al., 2015).

Synchrony, a central construct observed in the FFSF, refers to the temporal alignment of multimodal behaviors between infant and caregiver, distinct from contingency, which captures the directional responsiveness of one partner to the other. While contingency reflects a sequential, predictive relationship, synchrony emphasizes the bidirectional, co-occurring coordination of the dyad as a whole. This dynamic coordination supports emotional regulation and fosters the development of expectations about social interactions (Feldman, 2012a,b). Synchrony has been observed to increase during the Reunion phase compared to Play in the FFSF paradigm (Moore and Calkins, 2004).

The framework of bio-behavioral synchrony integrates behavioral and physiological processes to explain how caregiver-infant interactions scaffold co-regulation (Bell, 2020). Importantly, synchrony involves real-time, flexible adaptations rather than rigidly matched responses and it relies on the representation of temporal regularity, which makes the interaction partner more predictable (Hoehl et al., 2021).

The Still-Face episode is conceptualized as an experimental analog to everyday micro-stressors in caregiver-infant interactions. The infant's capacity to regulate distress and engage in reparative behavior throughout the FFSF is considered indicative of developing emotional regulation competencies. Indeed, the ability of the dyad to navigate and recover from interactive ruptures is predictive of later emotional and social development (Beebe et al., 2000; Müller et al., 2015; Tronick and Beeghly, 2011).

The FFSF has been widely employed in developmental research to investigate emotion regulation, stress physiology, and caregiver responsiveness (Mesman et al., 2009). Infant responses have been assessed through behavioral (Cassel et al., 2007; Toda and Fogel, 1993), physiological (Bazhenova et al., 2001; Montirosso et al., 2014), and neuroendocrine indexes, including heart rate, vagal tone (Moore and Calkins, 2004), skin conductance (Ham and Tronick, 2009), as well as cortisol and HPA axis reactivity (Ginnell et al., 2022; Haley and Stansbury, 2003; Lewis and Ramsay, 2005; Provenzi et al., 2016b;). Across studies, maternal sensitivity, seen as responsiveness to mismatches, emerges as a significant predictor of infant regulatory outcomes, with lower responsiveness linked to heightened stress markers during Reunion episodes (Haley and Stansbury, 2003; Moore and Calkins, 2004). Recent findings showed that maternal sensitivity predicts the coherence of an infant's cross-modal communicative signals, the coordinated use of multiple channels of communication simultaneously, such as gaze, facial expression, and vocalization; infants with sensitive mothers display more socially positive regulatory patterns and higher cooperation (Fuertes et al., 2024).

Disruptions in caregiver–infant mutual regulation may impede the co-construction of meaning and lead to maladaptive meanings and relational patterns (Tronick and Beeghly, 2011). In clinical contexts, the FFSF has proven valuable for identifying early signs of regulatory challenges and relational risk, particularly in dyads affected by maternal mental health issues or neurodevelopmental conditions (Giusti et al., 2018). It offers a structured, ecologically valid method for assessing co-regulation and guiding targeted interventions aimed at enhancing dyadic synchrony and emotional resilience.

## 4 Dyadic reparation and dyadic expansion of consciousness

**Figure 2:**
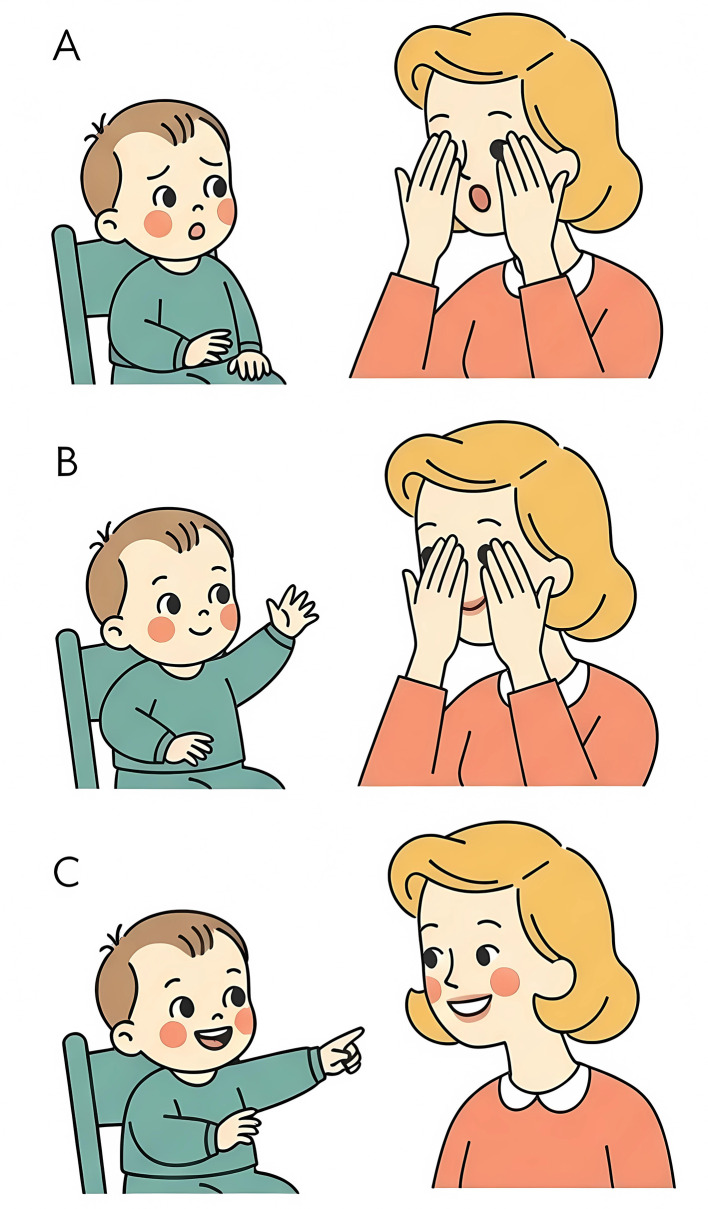
Graphical representation of dyadic expansion of consciousness. The mother plays “peek-a-boo” with the baby **(A)** and baby responds for the first time. The mother repeatedly plays “peek-a-boo” **(B)** and baby keeps the score of an interactive procedure that repeats through time. The mother and the infant expanded their individual state of consciousness into a dyadic one, allowing the infant to start the process activating a systemic pattern they both know and can reproduce to get reciprocal involvement and pleasure **(C)**.

Tronick introduced the concept of dyadic expansion of consciousness (DEC) as a hypothetical process underlying the intersubjective sense of “togetherness” that emerges during co-regulatory interactions (Davis, 2015; Tronick et al., 1998). The DEC hypothesis (see Figure 2) conceptualizes infants and caregivers as self-organizing systems, each capable of generating distinct states of consciousness. During interaction, the states of consciousness of caregiver and infant may become increasingly coherent and inclusive. In these moments, each partner integrates aspects of the other's physiological, emotional, cognitive, and behavioral states, forming a shared dyadic configuration that transcends each partner's individual state of consciousness (Tronick et al., 1998). Infants convey their internal states, and caregivers regulate and scaffold them, supporting higher neuropsychological organization. When this collaboration succeeds, it culminates in the emergence of a dyadic state, an integrated, co-created relational configuration that embodies enhanced complexity and coherence (Tronick, 2004; Tronick et al., 1998). Importantly, chronic absence of such dyadic experiences, as observed in contexts of institutional care, are meant to disrupt developmental trajectories. Deprivation of co-regulated interactions impairs the expansion of consciousness and may contribute to attachment disturbances and maladaptive relational patterns (Tronick, 2004). According to the DEC hypothesis, the failure to attain or maintain dyadic states of consciousness leads to a reduction in both coherence and complexity. This entropic process reflects diminished vitality, defined as the system's dynamic energy and responsiveness. It may manifest in flattened infant affect, reduced reciprocal engagement, and diminished integrative capacity-the caregiver-infant dyad's ability to coordinate physiological, emotional, cognitive, and behavioral states into a coherent relational pattern (Tronick, 2004).

Despite the theoretical richness of DEC, empirical research has largely concentrated on the behavioral and emotional aspects of dyadic reparation, with limited attention to the underlying neurophysiological mechanisms. Notable exceptions include research that explored peripheral neurophysiological indicators during reparation. For instance, Provenzi et al. (2015) examined vagal tone in 4-month-old infants, finding that higher vagal regulation supported better coping with social stress during mother-infant repair interactions. Similarly, Müller et al. (2015) investigated cortisol reactivity in infants, assessed via salivary cortisol as an index of stress. Their results indicated that successful dyadic repair is associated with attenuated physiological stress responses, emphasizing the regulatory role of repair in neuroendocrine functioning. However, these neurophysiological indicators reflect an individual's internal response to social stress or regulation. This provides a partial view of what is essentially a relational, two-person process. A significant gap remains in understanding how these individual regulatory efforts, such as the infant's vagal tone or the mother's cortisol levels, integrate into a shared neural system during the dance of interaction.

Emerging neuroscientific methodologies such as hyperscanning offer promising avenues to investigate the neural underpinnings of these co-regulatory processes. Hyperscanning allows for the simultaneous recording of brain activity in both caregivers and infants during real-time interactions, providing a unique opportunity to study the inter-brain dynamics that support reparation and mutual regulation. This technological advancement opens new perspectives for testing the DEC hypothesis and for deepening our understanding of the neural architecture of early intersubjectivity.

### 4.1 Hyperscanning: studying interacting brains on the go

Hyperscanning refers to the simultaneous recording of brain activity in two or more individuals engaged in real-time interaction (Babiloni and Astolfi, 2014; Provenzi et al., 2023). Originally introduced through functional magnetic resonance imaging (fMRI) (Montague et al., 2002), hyperscanning has since expanded to include several neuroimaging modalities, such as fMRI, functional near-infrared spectroscopy (fNIRS), electroencephalography (EEG), and magnetoencephalography (MEG) (Czeszumski et al., 2020). These techniques enable the investigation of interpersonal neural synchrony (INS) (Hoehl et al., 2025; Koul et al., 2023), a neurophysiological marker of dyadic coordination that reflects shared neural dynamics between interacting partners. In developmental research, hyperscanning is rapidly becoming a valuable tool for studying early social interactions, particularly parent–infant exchanges (Nguyen et al., 2020; Key et al., 2025) that are characterized by rapid transitions, and cycles of mismatch and repair. From this perspective, the choice of neuroimaging modality is theoretically based, since different techniques have different levels of sensitivity to the temporal, spatial and ecological properties of dyadic co-regulation. While fMRI has a high spatial resolution (3 mm), which makes it suitable for studying brain structure, it also features low temporal resolution (Glover, 2011). Its main limitation, the requirement for the subject to stay still inside a scanner, reduces its ecological validity, and makes it less suitable for investigating complex social interactions, such as caregiver-infant exchanges, in naturalistic settings (Czeszumski et al., 2020). Despite this, early studies utilized dual-scanner setups (Montague et al., 2002) or more recent developments like dual-head volume coils that allow for dyadic fMRI (dfMRI) acquisition (Babiloni and Astolfi, 2014; Lee et al., 2010, 2012). MEG provides excellent temporal resolution and high spatial resolution (milliseconds; Cohen, 1968), but it is costly and requires cryogenics and shielded rooms, limiting its use with infants (Holmes et al., 2023) Nevertheless, MEG's high resolution has encouraged the development of MEG hyperscanning to investigate real-time brain-to-brain interactions, such as mother-child dynamics (Hirata et al., 2014). Recently, wearable MEG has been developed by implementing optically pumped magnetometers (OPMs), which are sensitive magnetic field sensors that do not require cryogenics, allowing sensors to be placed closer to the scalp (Brickwedde et al., 2024; Brookes et al., 2022). However, despite the technology's potential for use in pediatric settings, including infants (Corvilain et al., 2025), studies in this specific area remain limited (Rhodes et al., 2024). Consequently, the integration of this innovative technique into hyperscanning research is still considered to be in an early and rapidly evolving developmental phase (Holmes et al., 2023).

The fNIRS and EEG methodologies are among the most frequently employed in developmental research due to their non-invasive nature and portability, which allow for greater ecological validity in naturalistic settings (Key et al., 2025; Turk et al., 2022a). As a cost-effective technique, fNIRS is resistant to motion artifacts (Nguyen et al., 2021a,b; Reindl et al., 2019; Scholkmann et al., 2013). Its main limitation is its low spatial resolution (1 cm), despite a relatively high temporal resolution (Wass et al., 2020). The EEG method, on the other hand, directly records electrical activity with an extremely high temporal resolution (millisecond scale), making it ideal for capturing the rapid micro-adjustments in moment-to-moment interactions (Koike et al., 2015). Although it has a low spatial resolution, its mobility and low operating cost have led to an increase in hyperscanning studies using mobile EEG in the developmental field (Leong et al., 2019; Santamaria et al., 2020; Wass et al., 2018; Xu et al., 2024). Mobile EEG systems offer additional flexibility by eliminating cables that might distract infants or limit movement, although these systems may have fewer electrodes and rely on dry sensors, which can present connectivity challenges (Turk et al., 2022a). Moreover, infant-specific EEG caps matched in electrode count to adult caps are necessary for reliable data acquisition in dual setups. While hyperscanning modalities offer complementary perspectives on dyadic interaction, their suitability depends on the theoretical framing of co-regulation. Models such as the mutual regulation model and dyadic expansion of consciousness emphasize rapid, non-linear interactional dynamics, which EEG is well suited to capture. Empirical evidence from EEG hyperscanning has already demonstrated its utility in identifying neural markers of joint attention and co-regulation in infants aged 4 to 12 months—a crucial window for the maturation of the ‘social brain' (Hoehl et al., 2025; Bánki et al., 2024). For instance, Wass et al. (2018) showed that during joint play, parental theta power closely tracked and responded to changes in infants‘ visual attention, suggesting that mature brains actively support and scaffold infants' attentional engagement during social interaction. Leong et al. (2017) demonstrated that direct gaze, compared to indirect gaze, enhances bidirectional neural synchrony between infants and adults in both theta and alpha bands during communication, suggesting that ostensive social signals act to bring infant and adult brains into mutual temporal alignment. Santamaria et al. (2020) found inter-brain networks were more interconnected in the alpha range (6-9 Hz) during maternal expression of positive relative to negative emotions.

By contrast, fNIRS is better suited to indexing slower hemodynamic changes associated with sustained engagement or affective attunement. Recent fNIRS-based studies showed that infant-adult neural coupling during naturalistic play is dynamically modulated by fluctuations in mutual gaze, joint attention, and speech prosody (Piazza et al., 2020), and inter-brain synchrony is higher during cooperation (Marriott Haresign et al., 2019). Table 1 shows that each neuroimaging modality has its advantages and disadvantages. The choice of technique should be based on the specific research question. EEG is one of the most suitable techniques for studying dyadic co-regulation of inter-brain coupling due to its high temporal resolution and portability. The fNIRS method seems comparable to EEG, with the difference that the signal is acquired indirectly and the temporal resolution is lower. Furthermore, the OPM-MEG technique appears to have even better characteristics than EEG, but it has not yet been studied widely or applied to parent-infant hyperscanning studies. To date, this technique has been used only with adults in hyperscanning studies (Holmes et al., 2023). In the present manuscript, EEG hyperscanning is therefore discussed as an illustrative methodological example for operationalizing dynamic systems models of co-regulation and dyadic expansion of consciousness at the neural level. This focus should not be interpreted as a normative or exclusive endorsement of EEG, as alternative techniques (e.g., fNIRS) remain highly valuable.

**Table 1:** Comparative overview of non-invasive neuroimaging techniques used in developmental hyperscanning studies.

Technique	Signal/brain activity	Temporal resolution	Spatial resolution	Mobility/ecological validity	Advantages for parent–infant hyperscanning
fMRI	Indirect (BOLD signal, hemodynamic response)	Second range	Millimeter range	Very low (participants must stay still in scanner)	(a) High spatial resolution; (b) Allows mapping of deep structures.
fNIRS	Indirect (oxy-/deoxy-hemoglobin contrast)	Second range	Centimeter range	High (portable, motion-tolerant)	(a) Suitable for naturalistic and developmental contexts; (b) Cost-effective.
EEG	Direct (electrical activity)	Millisecond range	Centimeter range	High (mobile systems)	(a) Excellent temporal resolution; (b) Prevalent method in literature.
MEG	Direct (magnetic fields from neural currents)	Millisecond range	Millimeter range	Low (requires shielded room, fixed head position)	(a) Excellent temporal and spatial resolution.
OPM-MEG	Direct (magnetic fields from neural currents)	Millisecond range	Millimeter range	Medium (more portable than MEG, but still requires shielded room)	(a) Excellent temporal and spatial resolution; (b) Allows head movements.

### 4.2 Brain-to-brain co-regulation indexes

Recent studies have advanced diverse methodological frameworks to analyze parent–infant EEG co-regulation, leveraging various connectivity metrics to capture the dynamic neural interplay during social interactions. These frameworks differentiate between concurrent (e.g., joint action, mutual gaze, mirroring, synchronous neural activity) and sequential/predictive (e.g., turn-taking, reciprocity, imitation) patterns of interaction (Wass et al., 2020). As such, both non-directed (simultaneous) and directed (time-lagged or causal) measures have been applied to explore the complex associations that characterize inter-brain communication.

Notably, methods used to quantify inter-brain EEG connectivity often parallel those developed for intra-brain analysis, relying on phase- and amplitude-based coupling measures to evaluate the degree and nature of neural alignment between individuals (Turk et al., 2022a). These include a range of synchrony indexes grounded in different assumptions about the temporal and spectral properties of EEG signals. Phase-based measures have been widely adopted to assess whether brain oscillations across dyads operate in stable temporal relationships. A central non-directed index is the Phase-Locking Value (PLV; Lachaux et al., 1999), which captures transient, frequency-specific phase alignment across brains. PLV values range from 0 (random phase relationship) to 1 (perfect coupling), and have been widely used in developmental EEG hyperscanning studies (Haresign et al., 2022; Santamaria et al., 2020). PLV builds on the broader assumption that consistent phase lags between signals reflect meaningful coupling—similar to within-brain synchronization dynamics (Fries, 2005; Van Mierlo et al., 2014). However, infant EEG is typically dominated by low-frequency activity, which diverges significantly from adult frequency bands (Marshall et al., 2002). This mismatch complicates direct synchrony analyses across dyads. Recent proposals suggest that cross-frequency coupling methods may offer a more nuanced strategy for analyzing interactional synchrony across developmentally distinct neurophysiological systems (Haresign et al., 2022; Noreika et al., 2020). Kayhan et al. (2022) showed cross-frequency PLV as an index of inter-brain phase alignement.

Other non-directed phase synchrony metrics include the weighted Phase Lag Index (wPLI), which reduces the influence of spurious or volume-conducted connections by focusing on non-zero phase lag relationships (Endevelt-Shapira et al., 2021), and Circular Correlation (CCorr), which quantifies the association of phase angles across oscillatory cycles in caregiver–infant dyads (Burgess, 2013). Coherence-based measures offer a joint assessment of power and phase similarity across time and frequency domains. Wavelet coherence and imaginary coherence, for example, provide time-resolved insights into the synchrony of neural signals at specific frequency bands and are particularly suited to analyzing transient, dynamic patterns of coordination in naturalistic interactions (Labat, 2005; Tafreshi et al., 2019). We hypothesize that DEC would manifest as a dynamic reorganization of inter-brain coupling during the Reunion phase of the FFSF, reflecting the dyad's adaptive flexibility following the Still-Face disruption. This prediction moves beyond a linear “more synchrony is always better” assumption, framing inter-brain coherence as a context-sensitive index of dyadic co-regulation (Gordon et al., 2025).

Considering that different participants may play different roles in many social interactions (e.g. the sender and receiver of information), there may be a time delay between two synchronous EEG signals (Xu et al., 2024). To address this asymmetry, directed measures seek to establish a statistical causation from the data, based on the assumption that causes precede their effects (Bastos and Schoffelen, 2016). Granger causality is one of the most common directed measures: this metric can be computed using a linear auto-regressive model fit to the data or through non-parametric spectral matrix factorization, and allows an estimation of directed interactions. In particular, it allows a sequential estimation of brain-to-brain coupling (Turk et al., 2022a) and a separate estimation of interaction from signal x to signal y, and from signal y to signal x (Bastos and Schoffelen, 2016; Granger, 1969). Partial Directed Coherence (PDC), based on the concept of Granger Causality, has emerged as a key approach (Turk et al., 2022a). PDC estimates causal relationships between signals using multivariate autoregressive (MVAR) modeling, thus extending traditional coherence by identifying the direction and strength of information flow (Ayrolles et al., 2021; Baccalá and Sameshima, 2001; Burgess, 2013; Leong et al., 2017).

Another measure is time-lagged cross-correlations, a time-domain approach for quantifying lagged inter-brain coupling (Wass et al., 2020). This method estimates the Pearson correlation between two neural signals across a range of temporal offsets and uses the highest correlation value as an index of their maximal temporal alignment (Xu et al., 2024). Time-lagged cross-correlation has been widely employed in neurophysiological research on social interaction to detect delayed dependencies between partners' neural activities (Brody, 1999; Kawasaki et al., 2013; Kuhlen et al., 2012).

The Phase Transfer Entropy (PTE) index of directed connectivity, despite not being widely used in developmental literature, allows researchers to measure sequential inter-brain synchrony. PTE has been defined as a measure of directed connectivity among neural oscillations, allowing detection of the strength and direction of connectivity even in the presence of noisy signals, such as those observed in EEG recordings (Lobier et al., 2014). Two-time series x and y are used to estimate transfer entropy (TE), comparing conditional probabilities. PTE has several advantages, including the fact that it is not based on the temporal structure of the data and that it can be computed over time and trials (Cohen, 2014; Haresign et al., 2022). In addition to phase and coherence metrics, amplitude-based measures offer another dimension for analyzing neural coupling (Ayrolles et al., 2021). Metrics such as Amplitude Envelope Correlation, Power Correlation, and Projected Power Correlation (PPC) capture co-variations in signal intensity, offering insight into shared fluctuations in neural activation across time (Dikker et al., 2021; Zamm et al., 2018; Zimmermann et al., 2024).

Taken together, these analytic approaches offer a diverse toolkit for investigating the multi-layered architecture of parent–infant brain-to-brain coordination. Rather than reflecting rigid alignment, synchrony in these contexts is increasingly conceptualized as a flexible, self-organizing process—one that integrates stability and variability to support adaptive co-regulation in complex, real-world interactions.

## 5 How hyperscanning can shed new light on the dyadic expansion of consciousness

Building on the caregiver–infant co-regulation literature, we propose that EEG hyperscanning offers a valuable methodological avenue for advancing research in early development (Nguyen et al., 2021a). Dual-brain neuroscientific approaches can reveal insights into behavioral processes central to mutual regulation—such as matching, synchrony, and reparation—which are foundational to children's emotional development. Applying hyperscanning within established theoretical models, such as Tronick's mutual regulation model (MRM; Tronick et al., 1998), may open new research directions and generate clinically meaningful evidence on the neural mechanisms underpinning dyadic co-regulatory behaviors and the process of dyadic expansion of consciousness (DEC).

The Face-to-Face Still-Face (FFSF) paradigm offers an ecologically grounded yet experimentally structured setting for investigating adult–infant brain activity across interactional phases. These include moments of behavioral matching, episodes of disruption (e.g., the Still-Face phase), and reparation processes during the reunion. Through this lens, hyperscanning enables exploration of how neural coordination supports, and is shaped by, real-time co-regulation within the dyad. As the paradigm consists of three episodes (Play, Still-Face, Reunion), dual-EEG data can be aligned with each episode to measure changes in brain-to-brain synchrony. Neural coordination during the Play episode may indicate matching and reciprocal engagement; the Still-Face episode offers a controlled window to investigate neural decoupling and the breakdown of mutual regulation; and the Reunion phase allows researchers to assess neural re-synchronization, providing a biomarker of dyadic reparation. The choice of analytical metrics—phase, coherence, amplitude-based, and directed connectivity measures—can be flexibly applied to capture neural dynamics associated with the distinct interactional features of each FFSF episode, depending on the specific research question. Thus, hyperscanning complements the well-validated behavioral and physiological indexes traditionally used in the FFSF and enhances its utility for studying mechanisms of co-regulation and developmental risk. While we agree that advancing specific predictions about metric behavior across phases is a valuable and necessary next step for the field, formulating a comprehensive set of quantitative hypotheses falls outside the scope of the present work. A dedicated methodological roadmap, including operationalization of metrics, control conditions, and optimal baselines, is required to specifically address these technical challenges.

A critical conceptual and methodological issue concerns how DEC can be differentiated from other forms of interpersonal coordination, such as shared attention, motor synchrony, or joint action. This distinction is particularly relevant in the context of the FFSF paradigm. Unlike naturalistic play settings that often involve external objects or instrumental caregiving routines (e.g., toy-mediated soothing or feeding), the FFSF intentionally excludes such elements. This constraint is not a limitation but a theoretically meaningful feature of the paradigm, as it isolates dyadic regulation processes that rely exclusively on mutual gaze, affective exchange, timing, and contingency, rather than on shared reference to external objects. From this perspective, EEG hyperscanning in the FFSF does not aim to capture neural correlates of object-based shared attention or motor entrainment. Instead, it offers a unique opportunity to investigate whether and how inter-brain coordination reflects internally generated, relationally grounded processes that persist beyond immediate behavioral coupling. If DEC is a meaningful construct at the neural level, we would not expect it to manifest as static or maximal inter-brain dynamics. Rather, DEC should be reflected in flexible, phase-dependent patterns of coupling, decoupling, and re-coupling across the Play, Still-Face, and Reunion episodes.

Specifically, the Reunion episode allows examination of neural re-synchronization processes following rupture. The temporal dynamics of this recovery (e.g., speed and organization of re-coupling) might potentially be investigated as neural proxies of dyadic reparation and, by extension, of DEC. Importantly, this framework implies that DEC is not indexed by the magnitude of inter-brain coupling *per se*, but by its temporal organization and adaptability across interactional states. In this sense, EEG hyperscanning in the FFSF paradigm is particularly well suited to disentangle dyadic expansion of consciousness from simpler forms of shared attention or motor coordination, precisely because such alternative mechanisms are experimentally minimized. In this sense, comparing the Reunion phase with the initial Play episode provides a crucial opportunity to disentangle the neural signatures of socio-emotional reparation from those of general joint engagement, allowing researchers to account for common behavioral artifacts present in both contexts.

One critical research question concerns the neural underpinnings of concurrent behavioral matching. How does behavioral alignment manifest at the neural level? One hypothesis is that shared attention to external stimuli, or mutual responsiveness to each other's actions, results in phase synchronization across brains. This may occur through mechanisms such as sensory entrainment (Attaheri et al., 2022; Lakatos et al., 2008; Power et al., 2012) or motor integration, whereby individuals align their internal states through coordinated movements (Koul et al., 2023; Pezzulo et al., 2013). Matching behaviors may also reflect convergent cognitive processing, which could give rise to synchronized amplitude patterns within particular frequency bands (Dikker et al., 2017). Specific frequency bands and scalp distributions can also inform on potential underlying mechanisms supporting brain co-regulation. However, it should be noted that the functional significance of neural oscillations in infancy differs substantially from that observed in adulthood. Caution is warranted when interpreting inter-brain synchrony measures (Marshall et al., 2002). Cross-frequency coupling approaches may offer a more appropriate framework for adult-infant dyads (Haresign et al., 2022; Kayhan et al., 2022; Noreika et al., 2020). With these caveats in mind, specific frequency bands may nonetheless inform on potential mechanisms of brain co-regulation. In particular, alpha band activity in infants has been linked to social processing and self-regulation mechanisms (Hofstee et al., 2022; Michel et al., 2015). In hyperscanning studies, alpha synchronization in mother-infant dyads has been associated with valence of the emotional feedback (Santamaria et al., 2020) and multisensory maternal stimulation (Neel et al., 2025). Frontal alpha asymmetry (FAA), which captures hemispheric differences in frontal alpha power and serves as a neural correlate of emotion regulation, has been assessed simultaneously in caregiver-infant dyads, showing that each partner's FAA responds reactively to the other's emotional signals during social interaction (Atzaba-Poria et al., 2017; Perone et al., 2020; Swider-Cios et al., 2024). Moreover, within the alpha band, the mu sub-band can be linked to action observation/production (Pineda, 2005) and interindividual coordination (Naeem et al., 2012). In hyperscanning studies decrease of signal power between 11 and 13 Hz over a large set of left centro-parieto-occipital electrodes has been linked to attention mirroring, social coordination, and mutual attentiveness (Lachat et al., 2012) and attention following in mother-child dyads (Hasegawa et al., 2016). Finally, theta activity in infants has been associated with sustained attention, socially relevant information processing (Begus and Bonawitz, 2024; Michel et al., 2024), and arousal (Orekhova et al., 2006). In hyperscanning studies, synchrony in this band has been associated with changes in shared attention during joint play (Wass et al., 2018) and maternal sensitive behavior (Endevelt-Shapira and Feldman, 2023). Theta activity in infants has been used to investigate leader-follower dynamics linking infant attentional and parental behaviors (Marriott Haresign et al., 2023; Phillips et al., 2023). Dual-brain recordings make it possible to link specific brain activity with behavior in real time while also assessing how two brains function together as a single, dynamic system. This approach enables researchers to study interpersonal processes not as isolated signals, but as integrated, co-evolving patterns across partners.

A second line of inquiry involves the temporal dynamics of parallel neural activity: how do interaction partners regulate and shift their brain states together, and to what extent is one partner influencing the other? For instance, mutual phase resetting around moments of shared attention (e.g., the onset of mutual gaze) may reflect the active shaping of shared neural states (Marriott Haresign et al., 2023) and enhance neural responses in both partners (Bánki et al., 2024). Changes in amplitude correlations, may indicate more complex joint action patterns, such as turn-taking or role alternation (Xu et al., 2024). Furthermore, indirect connectivity measures could provide insights into whether such synchrony is driven endogenously or partner-driven (Phillips et al., 2023), helping to distinguish between reactive and proactive regulatory dynamics.

A third research frontier concerns reparation following interactive rupture. In the FFSF, the Reunion episode provides an opportunity to examine neural re-coordination following a breakdown in interaction. One operational hypothesis is to measure how quickly and in what form dyads return to pre-disruption levels of brain synchrony. This “reparation lag” might vary across dyads and be associated with developmental outcomes. Alternatively, researchers could analyze evolving temporal patterns of neural activity that differentiate collaborative, reciprocal repair processes from unilateral or non-contingent responses. Turn-taking and behavioral co-regulation rhythms may generate temporally structured neural signals, reflecting higher-order dyadic coordination beyond simple realignment. While the operationalization of these neural patterns and their mapping onto specific analytical metrics is a critical next step currently under development, these neural dynamics may also relate to the emergence of jointness (Siposova and Carpenter, 2019) and the broader theoretical construct of dyadic expansion of consciousness. When partners are aware of each other's cognitive states and actions, a shared experience emerges—one that may be more than the sum of its parts.

Key questions remain: Can brain co-regulation indexes reveal a state of shared awareness? Or do they merely reflect coordinated activity without access to subjective experience? Is shared awareness an emergent property of the dyad, or does it remain an individual-level phenomenon interpreted intersubjectively? Investigating these questions demands careful attention to the complexity of neural co-regulation. Such dynamics unfold across multiple levels (i.e., temporal, spatial, spectral) and cannot be reduced to simplistic indicators. High synchrony values, for instance, should not be interpreted uncritically as signs of optimal dyadic interaction. Strong neural alignment may also reflect asymmetrical regulation, in which one partner (often the caregiver) exerts dominant influence while the other passively follows, scenarios that may limit opportunities for mutuality and infant-led engagement. Moreover, healthy interactions are marked by fluctuation, disruption, and recovery. Ruptures are not anomalies but essential features of adaptive relational processes. In sum, we suggest that dual-EEG hyperscanning holds promise not only for mapping moment-to-moment co-regulation but also for enriching developmental models of dyadic functioning. Embracing its full potential will require methodological precision, interpretative humility, and theoretical frameworks capable of capturing the non-linear, emergent, and embodied nature of caregiver–infant interaction.

### 5.1 Potential biases and warnings

Studying a theoretical construct as intricate as DEC through hyperscanning presents important conceptual, technical, ecological, and theoretical limitations. These challenges stem both from the complexity of the construct itself and the methodological characteristics of EEG hyperscanning. One major concern involves the risk of reductionism. The DEC hypothesis encompasses subjective, emotional, and intersubjective dimensions that resist straightforward operationalization through neurophysiological markers. There is currently no direct correspondence between EEG-derived indexes and the experiential or relational qualities theorized in DEC. Inter-brain synchrony measures may reflect aspects of shared activity or coordination, but they do not capture the full richness of emotional meaning-making, mutual awareness, or affective attunement. As such, EEG data should not be over-interpreted or taken in isolation. Instead, these neurophysiological indexes should be integrated into a biopsychosocial framework that also includes behavioral observations, emotional assessments, and relational dynamics (Hamilton, 2021). Hyperscanning may offer a window into the coherence of neural activity across brains (Hamilton, 2020); however, its findings must be interpreted with caution and in dialogue with existing theory.

On a technical level, EEG hyperscanning in caregiver-infant dyad faces considerable methodological constraints. The EEG signal is highly susceptible to motion artifacts, eye blinks, cardiac rhythms, and other noise sources, which are particularly difficult to control during naturalistic social interactions involving infants (Noreika et al., 2020). These issues can significantly compromise signal quality and reduce the reliability of observed effects. Furthermore, there is currently a lack of standardized preprocessing pipelines and analytic protocols designed for developmental hyperscanning data, leading to limited reproducibility and comparability across studies (Noreika et al., 2020; Turk et al., 2022a). While initiatives such as the DEEP pipeline (Kayhan et al., 2022) and the HyPyP pipeline (Ayrolles et al., 2021) offer promising structured approaches, broader adoption and consensus are still needed.

From an ecological standpoint, balancing experimental control with the naturalistic demands of real-world interaction remains a central challenge. The dynamic, multimodal nature of early caregiver-infant exchanges resists oversimplification, and tightly controlled lab protocols may fail to capture the complexity of spontaneous relational dynamics. At the same time, looser designs risk introducing extraneous variables that confound results. The variability in experimental paradigms and analytic decisions has thus limited the generalizability of findings and hindered the emergence of robust conclusions regarding inter-brain synchrony (Haresign et al., 2022; Kayhan et al., 2022; Noreika et al., 2020). Future progress will depend on striking a meaningful balance between ecological validity and methodological rigor.

To conclude, a theoretical concern. As hyperscanning studies yield increasingly fine-grained data, there is a risk of either forcing results into existing models without adequate fit or failing to adapt theoretical frameworks in response to new empirical evidence. Hyperscanning may challenge long-standing assumptions about interactional processes and open up previously unconsidered mechanisms, such as neural re-synchronization mechanisms following an interactive perturbation. Thus, ongoing conceptual refinement is essential. This requires sustained collaboration among neuroscientists, developmental psychologists, behavioral scientists, and clinicians to ensure that models of dyadic functioning remain responsive, integrative, and theoretically grounded. Despite these challenges, the dual-EEG method holds significant promise. It enables the investigation of socio-emotional development through the integration of multiple data modalities, such as synchronized video and audio recordings, physiological measures (e.g., heart rate), and micro-coded behavioral analysis, collected during authentic interactions (Turk et al., 2022b). The richness of these multi-layered data can enhance our understanding of the neurobehavioral foundations of DEC, provided that methodological, conceptual, and interpretive challenges are addressed through interdisciplinary effort. Adopting this complexity-oriented perspective, dual-EEG can be reframed not as an endpoint but as a starting point for exploring dyadic processes such as DEC. In doing so, we align with the principles of translational neuroscience, which views humans as intrinsically social beings whose development is grounded in early relational experiences (Provenzi et al., 2023). This approach underscores the necessity of moving beyond isolated brain-based measures to embrace a relational neuroscience of co-regulation and shared meaning-making from birth.

## 6 Conclusions

The mutual regulation model (MRM) and the concept of dyadic expansion of consciousness (DEC) (Tronick et al., 1998) have long provided the conceptual framework for understanding early caregiver–infant interactions. However, their empirical application has been limited by the historical absence of tools capable of capturing the neurophysiological underpinnings of these dynamic, relational processes. This manuscript offers both a theoretical discussion and methodological insights, highlighting the challenges and opportunities associated with examining a well-validated developmental framework through the lens of neuroscientific innovation. Future research would benefit from integrating EEG hyperscanning into developmental paradigms such as the face-to-face still-face (FFSF), guided by theoretical models capable of informing the interpretation of inter-brain data in early dyadic contexts.

## Data Availability

The original contributions presented in the study are included in the article/supplementary material, further inquiries can be directed to the corresponding author/s.
